# Assessment of the relationship between oral health-related quality of life (OHRQoL) and dental malocclusion in a Portuguese sample

**DOI:** 10.1080/07853890.2021.1896918

**Published:** 2021-09-28

**Authors:** Dinis Pereira, Vanessa Machado, João Botelho, Luís Proença, Ana Delgado, José João Mendes

**Affiliations:** a Egas Moniz Dental Clinic (EMDC), Instituto Universitário Egas Moniz (IUEM), Monte de Caparica, Portugal; b Centro de Investigação Interdisciplinar Egas Moniz (CiiEM), Egas Moniz – Cooperativa de Ensino Superior, C.R.L, Monte de Caparica, Portugal

## Abstract

**Introduction:**

The assessment of the oral health-related quality of life (OHRQoL) and dental malocclusion are described in the literature and the results indicate a decrease in OHRQoL when clinically determined dental malocclusion severity increase [1,2]. However, there is still a lack of information for the Portuguese population for this issue. This exploratory study aimed to evaluate the relationship between patient perception of OHRQoL and the severity of dental malocclusion in a Portuguese sample.

**Materials and methods:**

This work was approved by the Egas Moniz Ethics Committee. This cross-sectional observational study involved patients that sought orthodontic treatment between January and April 2019, at the Orthodontic Care Consultation of Egas Moniz Dental Clinic (Monte de Caparica – Almada, Portugal). Exclusion criteria were patients with severe diseases, craniofacial abnormalities, cognitive deficits, caries, periodontal diseases, and previous orthodontic treatment history. A total of 19 patients were enrolled in the study. OHRQoL was assessed by application of the Oral Health Impact Profile – Portuguese validated version (OHIP-14) [3] and dental malocclusion through the Index of Complexity, Outcome and Need (ICON) [4]. Based on the ICON score, patients were categorised as: in need of treatment (ICON > 43) or not (ICON ≤ 43). Resulting data were submitted to descriptive and inferential statistical analysis.

**Results:**

The sample included 7 (37%) males and 12 (63%) females, with a mean age of 27.9 years. Overall OHIP-14 score ranged from 0 to 49 and ICON score from 13 to 75 (9 (47.4%) subjects with ICON > 43 and 10 (52.6%) with ICON ≤ 43). Total OHIP-14 score and all seven median domain scores were not found to be significantly different (*p* = .113 to *p* = .968), when comparing both groups.

**Discussion and conclusions:**

OHRQoL was not found to be significantly different when considering the severity of dental malocclusion. Overall, results highlight a difference between patients’ self-perception of clinical condition impact in OHRQoL when compared to a clinical expertise judgement.
